# Effect of epidermal growth factor on cadherin-mediated adhesion in a human oesophageal cancer cell line.

**DOI:** 10.1038/bjc.1995.52

**Published:** 1995-02

**Authors:** H. Shiozaki, T. Kadowaki, Y. Doki, M. Inoue, S. Tamura, H. Oka, T. Iwazawa, S. Matsui, K. Shimaya, M. Takeichi

**Affiliations:** Department of Surgery II, Osaka University Medical School, Japan.

## Abstract

**Images:**


British Journal of Cancer (1995) 71, 250-258

© 1995 Stockton Press All rights reserved 0007-0920/95 $9.00

### Effect of epidermal growth factor on cadherin-mediated adhesion in a human oesophageal cancer cell line

H Shiozaki[1], T Kadowaki[1], Y Doki[1], M Inoue[1], S Tamura[1], H Oka[1], T Iwazawa[1], S Matsui[1], K Shimaya[1], M Takeichi[2] and T Mori[1]

[1]*Department of Surgery II, Osaka University Medical School, 2-2 Yamadaoka, Suita, Osaka 565, Japan;* [2]*Department of Biophysics, Faculty of Sciences, Kyoto University, Kitashirakawa, Sakyo-ku, Kyoto 606, Japan.*

**Summary** Epidermal growth factor (EGF) mediates many pleiotrophic biological effects, one of which is alteration of cellular morphology. In the present study, we examine the possibility that this alteration in cell morphology is caused in part by the dysfunction of cadherin-mediated cell-cell adhesion using the human oesophageal cancer cell line TE-2R, which expresses E-cadherin and EGF receptor. In the presence of EGF, TE-2R changed its shape from round to fibroblastic and its colony formation from compact to sparse. Vanadate, a tyrosine phosphatase inhibitor, further potentiated the EGF response, whereas herbimycin A, a tyrosine kinase inhibitor, interfered with it. Moreover, EGF enabled the cells to invade in organotypic raft culture. These phenomena were accompanied not by decreased expression of the E-cadherin molecule but by a change in its localisation from the lateral adhesion site to the whole cell surface. Both α- and β-catenin, cadherin-binding proteins, were also expressed at the same level throughout these morphological changes. Finally, we examined tyrosine phosphorylation of E-cadherin and α- and β-catenin, and observed tyrosine phosphorylation of β-catenin induced by EGF. These results suggest that EGF counteracts E-cadherin-mediated junctional assembly through phosphorylation of β-catenin and modulates tumour cell behaviour to a more aggressive phenotype.

**Keywords:** EGF; cadherin; β-catenin; cell-cell adhesion; tyrosine phosphorylation

Amplification of the epidermal growth factor receptor (EGFR) gene and consequent overexpression of EGFR have been observed in human carcinomas *in vivo* (Sainsbury *et al.*, 1985; Malden *et al.*, 1988; Yoshida *et al.*, 1989). In patients with squamous cell carcinomas of the oesophagus, we have previously demonstrated that overexpression of both EGFR and its ligand, transforming growth factor α (TGF-α), is associated with poor clinical outcome (Iihara *et al.*, 1993). Interestingly, overexpression of TGF-α EGFR is correlated more strongly with metastasis and invasion than with tumour size. EGFR transmits a mitogenic signal through its tyrosine kinase activity. However, activation of EGFR also influences a number of other phenotypic properties in malignant cells *in vitro*, including cell motility stimulation (Lund-Johansen *et al.*, 1990) and matrix protease production (Boyd, 1989; Niedbala and Sartorelli, 1989). Consequently, acceleration of cell motility and proteolysis is considered to cause invasion when EGFR is activated. On the other hand, we have demonstrated that intercellular adhesion mediated by E-cadherin is another major factor which restricts cell invasion and metastasis (Doki *et al.*, 1993; Oka *et al.*, 1993). Therefore, in this study, we explore the possibility that EGF EGFR perturbs the E-cadherin adhesion system and might consequently induce tumour invasion.

Cell-cell adhesion is mainly regulated by homotypic interaction of cadherin molecules, which are anchored to the cytoskeleton via associated cytoplasmic proteins, such as α- and β-catenin (Nagafuchi and Takeichi, 1988; Ozawa *et al.*, 1989) and the 220 kDa protein (Itoh *et al.*, 1991). Thus, cadherin-mediated cell adhesion is composed of many components and could be disrupted in a variety of ways. Three mechanisms of alteration of the cadherin mediated cell-cell adhesion system have been described in human cancer *in vivo* and *in vitro*. The first is down-regulation of cadherin expression (Behrens *et al.*, 1989; Frixen *et al.*, 1991) and the second is deletion of α-catenin (Shimoyama *et al.*, 1992). In the case of oesophageal cancer, reduced expression of either E-cadherin or α-catenin has been detected in 80% of tumours (Kadowaki *et al.*, 1994). The third abnormality of this adhesion system is biochemical modification of catenins. Tyrosine phosphorylation of catenins suppresses cadherin function *in vitro* (Matsuyoshi *et al.*, 1992; Behrens *et al.*, 1993; Hamaguchi *et al.*, 1993). Since, in these experiments, tyrosine phosphorylation was induced by v-Src, a non-receptor type tyrosine kinase, it would be interesting to analyse the effect of receptor tyrosine kinase activation on E-cadherin-mediated cell-cell adhesion.

In this study, we used TE-2R cells, an E-cadherin-positive clone from human oesophageal cancer TE-2 (Doki *et al.*, 1993), and found alteration of cell morphology and adhesiveness in the presence of EGF, which is accompanied by tyrosine phosphorylation of β-catenin. These results are described and the possible action of EGF EGFR in cell adhesion and invasion is demonstrated.

### Materials and methods

### *Cell culture*

TE-2R and TE-2S were cloned by the limiting dilution method from TE-2, a poorly differentiated squamous cell carcinoma of the human oesophagus (kindly provided by Dr Nishihira, University of Tohoku, Japan), as previously described (Doki *et al.*, 1993). Cells were maintained in a 1:1 mixture of Dulbecco's modified Eagle medium and Ham's F12 medium supplemented with 5% fetal calf serum (DH5).

### *Antibodies and growth factor*

The following antibodies were used in this study: mouse MAb against human E-cadherin (HECD-1) (Shimoyama *et al.*, 1989), rat MAb against α-catenin (α-18) provided by Drs S Tsukita and A Nagafuchi, rabbit polyclonal antibody against β-catenin provided by Dr S Shibamoto, mouse MAb against human EGFR (Ab-1, Oncogene Science, Mineola, NY, USA) and mouse MAb against phosphotyrosine (4G10, Upstate Biotechnology, Lake Placid, NY, USA) Human

Correspondence: H Shiozaki, Department of Surgery II, Osaka University Medical School, 2-2 Yamadaoka, Suita City, Osaka 565, Japan
Received 13 July 1994; revised 27 September 1994; accepted 30 September 1994

recombinant EGF was purchased from Gibco BRL. (Grand Island. NY. USA).

*Immunofluorescent cytochemistry*

Cells cultured on 15 mm coverslips were fixed with 3.5% paraformaldehyde in phosphate-buffered saline (PBS) for 30 min at 4°C. extracted with methanol at −20°C for 10 min. rinsed with 0.01 M Tris-buffered saline (pH 7.2) containing 1 mM calcium chloride (TBS-Ca). treated with 5% skimmed milk. or 1% bovine serum albumin for phosphotyrosine for 30 min and subsequently incubated with HECD-1 or 4G10 for 60 min at room temperature. After extensive washing with TBS-Ca. the samples were incubated with fluorescein isothiocyanate (FITC)-labelled anti-mouse IgG (Cappel. Durham. NC. USA). After washing. the preparations were mounted with Permafluor aqueous mounting medium (Immunon. Pittsburgh. PA. USA) to prevent bleaching. They were examined and photographed using a phase-contrast fluorescence microscope (Olympus. Tokyo. Japan). To examine the effect of EGF. cells were incubated in the presence of 30 ng $ml^{-1}$ EGF with or without 0.5 mM vanadate or 0.2 μg $ml^{-1}$ herbimycin A for 30 min. In order to induce extensive morphological changes. treatment with EGF was continued for 24 h. The cells were then subjected to immunofluorescent cytochemistry as described above.

*Immunoprecipitation*

To detect E-cadherin and cadherin-associated proteins by immunoprecipitation. $4 \times 10^6$ cells were lysed with 2 ml of the extraction buffer (1% Triton X-100. 1% Nonidet P-40. 1 mM calcium chloride. 2 mM phenylmethylsulphonyl fluoride and 20 μg $ml^{-1}$ leupeptin in 50 mM TBS pH 7.4) and centrifuged at 12 000 r.p.m. for 30 min. The supernatant was preabsorbed by incubation with 50 μl of protein A-Sepharose (Pharmacia LKB Biotechnology. Uppsala. Sweden) for 30 min. After removing the beads. the solution was mixed with 50 μg of HECD-1 for 2 h and then incubated with 100 μl of protein A-Sepharose for 2 h. The beads were collected by centrifugation. washed five times with extraction buffer. then suspended in 100 μl of SDS sample buffer containing 5% 2-mercaptoethanol. and boiled for 5 min. The released materials were analysed by immunoblotting. Stimulation by EGF was performed in the same way as immunofluorescent cytochemistry.

*Immunoblotting*

Immunoblot analysis was carried out as described previously (Nose and Takeichi. 1986) with some modifications. Briefly. $1 \times 10^6$ cells were lysed with 100 μl of sample buffer containing 2% SDS. After boiling for 5 min in the presence of 5% 2-mercaptoethanol. 10 μl of the sample was subjected to separation on a polyacrylamide gradient gel SDS-PAGE 4 20 (Daiichi Pure Chemical. Tokyo. Japan). The fractionated proteins were transferred onto a nitrocellulose membrane. After blocking for 1 h with 5% skimmed milk. or with 1% bovine serum albumin for phosphotyrosine. the membranes were incubated with HECD-1 or other antibodies for 1 h at room temperature. Visualisation was performed using an Immune-Blot Assay Kit (Bio-Rad. Hercules. CA. USA).

*Cell aggregation assay*

Cadherin-dependent cell aggregation was assayed as described before (Takeichi. 1977). Briefly. cells were treated with 0.01% crystallised trypsin in the presence of 0.5 mM calcium chloride at 37°C for 20 min. and then washed with $Ca^{2+}$- and $Mg^{2+}$- free 4-(2-hydroxyethyl)-1-piperazineethanesulphonic acid-buffered (pH 7.4) Hanks' balanced salt solution to obtain single-cell suspensions. Cells suspended in this solution containing 0.3 mM calcium chloride with or without 30 ng $ml^{-1}$ EGF were placed in wells of a 24-well plate and incubated to allow aggregation for 30 min at 37°C on a gyratory shaker rotating at 80 r.p.m. The aggregates were observed and photographed using a phase-contrast microscope (Olympus. Tokyo. Japan). For further culturing. cells were collected and resuspended in 2 ml of DH5 with the same concentration of EGF. put on the 3.5 cm plastic dish coated with 1% agar to prevent cells adhering to the bottom and incubated in 5% carbon dioxide incubator for 48 h.

*Cell dissociation assay*

Cell dissociation was measured quantitatively using the Transwell system (pore size 12 μm) (Costar. Cambridge. MA. USA). as previously described (Doki *et al*.. 1993). Approximately $1 \times 10^5$ cells were trypsinised and suspended in 500 μl of DH5. The cell suspensions were then applied to the upper compartment. and the lower compartment was filled with 1500 μl of DH5. After incubation for 24 h. the upper compartment was placed into a fresh lower compartment and the media in both were exchanged with DH5 containing graded doses (0-30 ng $ml^{-1}$) of EGF. Following a 48 h incubation. the cells. which passed through the micropore membrane and adhered to the bottom of the lower compartment were fixed with 10% formaldehyde. stained with haematoxylin and counted using a microscope.

*Invasion assay with organotypic raft culture*

*In vitro* tumour invasiveness was evaluated using organotypic raft culture. as previously described (Doki *et al*.. 1993). Briefly. in a 12-well plate. $2.5 \times 10^5$ tumour cells resuspended in 1 ml of medium were seeded on the gel of DH5 containing 1.0 mg $ml^{-1}$ type I collagen (Cell Matrix Type I-A. Nitta Gelatin, Osaka, Japan) and $3 \times 10^5$ human lung fibroblasts of the MRC-5 cell line (provided by the Japanese Cancer Research Resources Bank). After incubation for 24 h. gels were detached from the well. incubated for 24 h to induce contraction of the gel and floated at the air-fluid interface on stainless-steel grids placed into 100 mm culture dishes. Cells were refed every other day with 10 ml of DH medium containing 10% fetal calf serum with or without EGF at 10 ng $ml^{-1}$ and 30 μg $ml^{-1}$ in HECD-1. After 14 days. the composite gels were fixed with 10% formaldehyde. paraffin embedded. sectioned and stained with haematoxylin and eosin. They were observed and photographed using a microscope.

### Results

*Morphological changes by EGF in two-dimensional culture*

Immunoblot analysis using total cell lysates revealed that the TE-2R cells expressed a considerable amount of EGFR. although the level was found to be lower than in A431 cells (Figure 1a). Upon addition of EGF. autophosphorylation of the EGFR was apparent by immunoblot analysis using an anti-phosphotyrosine antibody (Figure 1b). We then examined the effect of EGF on cell growth and found that EGF slightly suppressed growth in a dose-dependent manner (0-100 ng $ml^{-1}$). After 7 days' incubation. a maximum 30% suppression of growth was observed in the presence of 100 ng $ml^{-1}$ EGF as compared with control untreated cells (data not shown). TE-2R cells expressed a large amount of E-cadherin (Figure 1c) and also expressed both α- and β-catenin. which bind the cytoplasmic domain of cadherin and regulate cadherin function (Figure 1d and e).

A striking morphological change in TE-2R was observed after 24 h of exposure to EGF. More than 10 ng $ml^{-1}$ EGF led to an extensive change in cellular morphology. but at concentrations greater than 100 ng $ml^{-1}$ EGF appeared slightly toxic for cell growth. Therefore. in the following assay. we chose to treat cells with 30 ng $ml^{-1}$ unless otherwise indicated. TE-2R cells originally exhibited a round cell shape and formed cobblestone-patterned colonies. Following

EGF treatment, TE-2R cells exhibited a fibroblastic shape and a dispersion of colony formation (Figure 2a and b). To examine whether this alteration of cellular morphology is linked with the action of the tyrosine kinase, we tested the effect of vanadate, an inhibitor of phosphotyrosyl protein phosphatases that consequently potentiates tyrosine phosphorylation (Brown and Gorden, 1986). Medium containing EGF and 0.5 mM vanadate was added to TE-2R cells and led to a further alteration in cell morphology (Figure 2c). We next examined the effect of herbimycin A, a specific inhibitor of tyrosine kinase (Uehara *et al.*, 1988). The addition of 0.2 $\mu g\ ml^{-1}$ herbimycin A inhibited the change in cell morphology induced by EGF (Figure 2d).

To investigate whether this morphological change induced by EGF involved cell–cell adhesion mediated by E-cadherin, we stained TE-2R cells with HECD-1, an antibody for human E-cadherin. In the absence of EGF, the edge of cell–cell contact sites overlapped each other with jagged

**Figure 1** **a**, Immunoblot analysis using total cell lysates of EGFR (MW = 175 kDa) **b–e**, Tyrosine phosphorylation 10, 30 or 60 min after treatment with (**b**) 30 ng $ml^{-1}$ EGF, (**c**) E-cadherin (E-cad, MW = 124 kDa) (**d**) α-catenin (α-cat, MW = 102 kDa) and (**e**) β-catenin (β-cat, MW = 88 kDa). Lane 1, A431 cells; lanes 2, TE-2R cells; lanes 3, TE-2S cells (negative control; E-cadherin negative clone). Molecular weight markers of 205, 116.5 and 80 kDa are indicated by bars on the left.

**Figure 2** Effect of EGF, vanadate, and herbimycin A on cell morphology after 24 h of exposure in TE-2R cells. **a**, No treatment. **b**, In the presence of 30 ng $ml^{-1}$ EGF. **c**, In the presence of 30 ng $ml^{-1}$ EGF and 0.5 mM vanadate. **d**, In the presence of 30 ng $ml^{-1}$ EGF and 0.2 $\mu g\ ml^{-1}$ herbimycin A.

surfaces, where E-cadherin was strongly stained (Figure 3a). Apparent changes in E-cadherin distribution were observed much earlier than major changes in cell morphology. After 30 min incubation with EGF, immunoreactive lines that segregate individual cells became sharp and smooth; in addition, a small amount of E-cadherin was detected in the apical surface of the cell membrane (Figure 3b). Treatment of cells with vanadate enhanced the staining pattern whereby more E-cadherin was observed in the apical cell surface. Moreover, intercellular adhesions were partially disrupted and E-cadherin was stained as a non-continuous line (Figure 3c). Cells treated with EGF and herbimycin A displayed the same E-cadherin localisation as untreated cells (Figure 3d). After incubation for 24 h with EGF, most E-cadherin existed on the apical surface and a small amount remained in the cell-cell contact sites (Figure 3e). Although an extensive alteration of E-cadherin localisation was induced by EGF, the total fluorescence of E-cadherin staining appeared to be at similar levels throughout these experiments.

We also performed immunofluorescent staining utilising 4G10, an antibody against phosphotyrosine residues. We did not observe any obvious differences after 24 h treatment with EGF. However, in the early phase of EGF treatment, significant changes were observed. Figure 3f demonstrates that in untreated TE-2R cells phosphotyrosine staining was weakly detected in focal contacts. In EGF-treated cells, phosphotyrosine stained predominantly in sites of cell–cell contact and not in sites of focal contact (Figure 3g). In the presence of both EGF and vanadate, phosphotyrosine staining in sites of cell–cell contact was enhanced (Figure 3h). In contrast, addition of herbimycin A to EGF-treated cells led to a phosphotyrosine pattern that resembled untreated cells (Figure 3i).

*Effect of EGF on cell adhesive capacity*

The cell–cell binding activity was evaluated by both cell aggregation and dissociation assays. In the cell aggregation assay, cell aggregates were observed in TE-2R cell culture after a 30 min incubation period (Figure 4a). E-cadherin was found to be responsible for this aggregation since an antibody against E-cadherin blocked this response (Doki *et al.*, 1993). In the 30 min time period, EGF did not inhibit the aggregation of TE-2R cells (Figure 4b). However, since it took much more time to induce obvious morphological change in two-dimensional cutlure, we extended the time in suspension culture on agar to 48 h. Within this time period, the aggregates of TE-2R cells became compact with smooth surfaces and the cells flattened and adhered tightly (Figure 4c), whereas in EGF-treated TE-2R cells such compaction was not induced and cell remained round and loosely adherent (Figure 4d).

Cell dissociation was evaluated by counting the cells that passed through a micropore membrane after 48 h of incubation. In our previous study, the dissociation of TE-2R cells was facilitated by disruption of cell–cell adhesion using an E-cadherin antibody (Doki *et al.*, 1993). EGF also strongly facilitated the dissociation of TE-2R cells in a dose-dependent manner (Figure 5).

*Effect of EGF on invasion assays* in vitro

Organotypic raft culture was performed to assess the effect of EGF on invasiveness *in vitro*. TE-2R cells did not possess invasive capacity, however cell invasion was induced upon addition of HECD-1 (30 $\mu g\ ml^{-1}$) (Figure 6a and b), as previously described (Doki *et al.*, 1993). In the presence of EGF (10 $ng\ ml^{-1}$), TE-2R cells displayed invasive capacity, although the invading cell cluster was larger and the depth of invasion was shallower than that treated with HECD-1 (Figure 6c). In cooperation with HECD-1, EGF facilitated invasion most strongly, and disruption of stratified epithelium was also observed (Figure 6d).

*Molecular mechanism of dysfunction in the E-cadherin adhesion system*

Although, the effect of EGF on the E-cadherin adhesion system was considered to be partly responsible for the morphological change and down-regulation of adhesive capacity shown in the above experiments, the molecular mechanism remained to be elucidated. At first, we examined the amount of E-cadherin and α- and β-catenin in total cell extracts by immunoblotting. No difference was observed in the expres-

**Figure 3** Immunofluorescent staining for E-cadherin (**a**–**e**), and phosphotyrosine (**f**–**i**), in TE-2R cells. **a** and **f**, No treatment. **b**, **h** and **g**, In the presence of 30 ng ml$^{-1}$ EGF after 30 min. **e**, In the presence of 30 ng ml$^{-1}$ EGF after 24 h. **c** and **h**, In the presence of 30 ng ml$^{-1}$ EGF and 0.5 mM vanadate after 30 min. **d** and **i**, In the presence of 30 ng ml$^{-1}$ EGF and 0.2 μg ml$^{-1}$ herbimycin A after 30 min.

**Figure 4** Cell aggregation assay of TE-2R cells. Aggregation after 30 min incubation **a** and **b** and on agar for 48 h **c** and **d** without **a** and **c** or with **b** and **d** 30 ng ml$^{-1}$ EGF.

**Figure 5** Cell dissociation assay. The number of cells passing through the micropore membrane in 48 h is indicated. The effect of EGF concentration was evident in TE-2R cells (■), but not in TE-2S cells (□) (negative control; E-cadherin-negative clone).

sion of the three molecules between EGF-treated and untreated TE-2R cells (Figure 7a–c).

It is well known that E-cadherin and α and β-catenin form a complex which is stable against some detergents and in co-precipitation with an E-cadherin antibody (Ozawa *et al.*, 1989). In TE-2R cells, these three molecules were detected by immunoblotting the immune complex of HECD-1, and it was observed that the amount and proportion of the three molecules did not change in the presence of EGF (data not shown). Finally, we examined tyrosine phosphorylation of the immune complex using the 4G10 antibody, which specifically recognises phosphotyrosine residues. In the absence of EGF, none of the components of the immune complex was tyrosine phosphorylated. However, in the presence of EGF, the 4G10 antibody revealed an 88 kDa protein, which was confirmed to be β-catenin by immunoblotting (Figure 7d). Moreover, phosphorylation of the 88 kDa band of β-catenin was strongly enhanced by the addition of vanadate and disappeared upon addition of herbimycin A to EGF-treated TE-2R cells (Figure 7d).

### Discussion

The present study describes the effect that EGF has on the E-cadherin adhesion system and the consequent effects on cellular morphology. In the presence of EGF, TE-2R cells displayed morphological changes including transition to a fibroblastic cell shape and dispersed colony formation, which was similar to that of the cells without E-cadherin (Doki *et al.*, 1993). Also, in long-term aggregation and cell dissociation assays, the adhesive capacity of TE-2R cells was suppressed by EGF. These results suggest that, in the presence of EGF, TE-2R cells lose their characteristic morphology, accompanied by changes to E-cadherin expression.

Striking changes were not observed, however, in short-term aggregation or morphology assays during the first 30 min of exposure to EGF, and it took more than 24 h to induce obvious changes. The process of forming complete cell–cell adhesion by cadherin was explained by Takeichi (1977) as follows. At first, cadherins are distributed over the whole cell

**Figure 6** Invasion assay of TE-2R cells in organotypic raft culture. **a**, No treatment. **b**, In the presence of 30 $\mu$g ml$^{-1}$ HECD-1. **c**, In the presence of 10 ng ml$^{-1}$ EGF. **d**, In the presence of 10 ng ml$^{-1}$ EGF and 30 $\mu$g ml$^{-1}$ HECD-1.

**Figure 7** Immunoblot analysis of (**a**) E-cadherin (E-cad, MW = 124 kDa) in total cell extracts (lanes 1 and 2), detergent-soluble fraction (lanes 1s and 2s) and insoluble fraction (lane 1i and 2i), (**b**) α-catenin in total cell extracts (α-cat, MW = 102 kDa), (**c**) β-catenin in total cell extracts (β-cat, MW = 88 kDa) and (**d**) tyrosine phosphorylation in immunoprecipitated E-cadherin/catenin complexes of TE-2R cells. Lane 1, no treatment; lane 2, in the presence of 30 ng ml$^{-1}$ EGF; lane 3, in the presence of 30 ng ml$^{-1}$ EGF and 0.5 mM vanadate; lane 4, in the presence of 30 ng ml$^{-1}$ EGF and 0.2 $\mu$g ml$^{-1}$ herbimycin A.

surface in isolated cells. When two cells come into contact, they are bound by cadherin at one point. Thereafter, other cadherins concentrate at this point, the shape of the cells is deformed and a large area of cell–cell contact forms. This last phenomenon is designated compaction and requires catenins and association with the cytoskeleton. Short-term aggregation assays mainly detect the first step in the cadherin reaction, and the degree of aggregation depends on the number of cadherin molecules'. EGF suppressed not short-term aggregation but long-term compaction, therefore EGF was considered to affect some aspect of this sequence of events. This assumption is consistent with the observation that E-cadherin is distributed over the whole cell surface after 24 h of exposure to EGF.

EGF transmits its signal to the cell via the EGFR, a member of the receptor-type tyrosine kinase family, which is activated by autophosphorylation of its tyrosine residues. Both EGFR and E-cadherin are known to localise in intercellular adherence junctions (Fukuyama and Shimizu, 1991). Immunoblotting and immunostaining by a phosphotyrosine antibody revealed that EGFR was located at cell contact sites and was strongly autophosphorylated by stimulation with EGF. This led us to believe that the tyrosine kinase activity of EGFR was very closely associated with the E-cadherin adhesion system. In fact, vanadate, a tyrosine phosphatase inhibitor, potentiated the effect of EGF on the E-cadherin adhesion system. In contrast, herbimycin A, a tyrosine kinase inhibitor, reduced this effect. These results imply that tyrosine phosphorylation of a member of the E-cadherin system is involved in the action of EGF. It has been shown that activation of EGFR leads to phosphorylation of several cytoskeleton-associated proteins, such as ezrin, vinculin and spectrin (Akiyama *et al.*, 1986; Bretscher, 1989). Although interaction between these cytoskeletal proteins and cadherin is not understood, the E-cadherin adhesion system might be phosphorylated in a similar way.

Thus, tyrosine phosphorylation of the E-cadherin system seemed to be partly responsible for the morphological change induced by EGF. Therefore, we examined tyrosine phosphorylation of E-cadherin and α- and β-catenin and found tyrosine phosphorylation of β-catenin in EGF-treated TE-2R cells. It has recently been reported that tyrosine phosphorylation of the cadherin catenin complex suppresses cadherin function *in vitro* (Matsuyoshi *et al.*, 1992; Behrens *et al.*, 1993; Hamaguchi *et al.*, 1993).

Although β-catenin forms complexes with cadherin and α-catenin, its exact role remains to be elucidated. Experiments utilising cells expressing fusion proteins of cadherin and α-catenin that lack β-catenin binding sites demonstrate that these cells adhere to each other more tightly than cells with normal cadherins (S Tsukita, unpublished data). β-Catenin might have the effect of down-regulating cadherin function. Recently, binding between the APC tumour-suppressor gene product and β-catenin was reported, and it was suggested that mutation of APC might render inadequate the binding capacity between APC and β-catenin (Rubinfeld *et al.*, 1993; Su *et al.*, 1993). The association between APC and phosphorylation of β-catenin is not clear and should be resolved in the future.

In organotypic raft culture, EGF-treated TE-2R cells invaded the gel. Tumour invasion involves many factors, including proteolysis and cell motility, which are stimulated by EGF (Engström 1986; Boyd, 1989; Niedbala and Sartorelli, 1989). In this study, we indicate the possibility that down-regulation of E-cadherin by EGF might be another major factor that facilitates cell invasion.

We have studied E-cadherin and α-catenin expression in carcinomas *in vivo* and found that decreased expression is significantly correlated with dedifferentiation, invasion and metastasis (Kadowaki *et al.*, 1994; Matsui *et al.*, 1994). However, some tumours, especially signet ring cell carcinomas of the stomach, express both E-cadherin and α-catenin and exhibit inconsistent invasion, forming dispersed colonies (Matsui *et al.*, 1994). Some of them have been shown to have a mutated E-cadherin gene (Becker *et al.*, 1994; Oda *et al.*, 1994), but the rest are supposed to have intact E-cadherin genes and associated proteins. On the other hand, some diffuse types of gastric cancers, including signet ring cell carcinomas, are known to express many unidentified proteins with enhanced phosphorylation of tyrosine residues (Takeshima *et al.*, 1991). These phenomena might have some association with tyrosine phosphorylation of β-catenin.

However, it is not known what type of tyrosine kinase can induce the tyrosine phosphorylation of β-catenin *in vivo*. *In vitro*, the association of EGFR was suggested in the present study, and v-Src has been suggested in the previous studies (Matsuyoshi *et al.*, 1992; Behrens *et al.*, 1993; Hamaguchi *et al.*, 1993). We observed similar morphological changes and phosphorylation of β-catenin in other squamous cell carcinomas (T-T, TTn, TE-3; authors' unpublished observation). All of them overproduce EGFR, so that EGFR might be the most plausible candidate in oesophageal squamous cell carcinoma. Furthermore, Gavrilovic *et al.* (1990) have reported that TGF-α, an EGFR ligand, induces a motile fibroblast-like phenotype *in vitro*. This study supports our hypothesis. However, various growth factor receptors with tyrosine kinase activity have been reported to be associated with carcinogenesis and tumour progression *in vivo* (Ullrich and Schlessinger, 1990). Hepatocyte growth factor receptor is one of them and is known to promote not only cell growth but cell scattering (Matsumoto and Nakamura, 1992), indicating that phosphorylation of β-catenin may be involved. Thus, the association of growth factor signal transduction and cell adhesion is an important problem that should be further investigated.

**Acknowledgements**
The critical reading of Ms AM Cacace and the technical assistance of Ms Y Naito are gratefully acknowledged. This study was supported by Grants-in-Aid for Cancer Research from the Ministry of Education, Science and Culture (No. 06281239) and the Ministry of Health and Welfare (No. 5-12), Japan.

**References**

AKIYAMA T, KADOWAKI T, NISHIDA E, KADOOKA T, OGAWARA H, FUKAMI Y, SAKAKI H, TAKAKU F AND KASUGA M. (1986). Substrate specificities of tyrosine-specific protein kinases towards cytoskeletal proteins *in vitro*. *J. Biol. Chem.*, **261**, 14797–14803.

BECKER KF, ATKINSON MJ, REICH U, BECKER I, NEKARDA H, SIEWERT JR AND HÖFLER H. (1994). E-cadherin gene mutations provide clues to diffuse type gastric carcinomas. *Cancer Res.*, **54**, 3845–3852.

BEHRENS J, MAREEL MM, VAN ROY FM AND BIRCHMEIER W. (1989). Dissecting tumor cell invasion: epithelial cells acquire invasive properties after the loss of uvomorulin-mediated cell–cell adhesion. *J. Cell Biol.*, **108**, 2435–2447.

BOYD D. (1989). Examination of the effects of epidermal growth factor receptor on the production of urokinase and the expression of the plasminogen activator receptor in a human colon cancer cell line. *Cancer Res.*, **49**, 2427–2432.

BEHRENS J, VAKAET L, FRIIS R, WINTERHAGER E, VAN ROY F, MAREEL MM AND BIRCHMEIER W. (1993). Loss of epithelial differentiation and gain of invasiveness correlates with tyrosine phosphorylation of the E cadherin β-catenin complex in cells transformed with a temperature-sensitive v-src gene. *J. Cell Biol.*, **120**, 757–766.

BRETSCHER A. (1989). Rapid phosphorylation and reorganization of ezrin and spectrin accompany morphological changes induced in A431 cells by epidermal growth factor. *J. Cell Biol.*, **108**, 921–930.

BROWN DJ AND GORDEN A. (1986). The stimulation of $pp60^{v\text{-}src}$ kinase activity by vanadate in intact cells accompanies a new phosphorylation state of the enzyme. *J. Biol. Chem.*, **259**, 9580–9586.

DOKI Y, SHIOZAKI H, TAHARA H, INOUE M, OKA H, IIHARA K, KADOWAKI T, TAKEICHI M AND MORI T. (1993). Correlation between E-cadherin expression and invasiveness in vitro in a human eosphageal cancer cell line. *Cancer Res.*, **53**, 3421–3426.

ENGSTRÖM W. (1986). Differential effects of epidermal growth factor on cell locomotion and cell proliferation in a cloned human embryonal carcinoma-derived cell line. *J. Cell. Sci.*, **86**, 47–55.

FRIXEN UH, BEHRENS J, SACHS M, EBERLE G, VOSS B, WARDA A, LÖCHNER D AND BRICHMEIER W. (1991). E-cadherin-mediated cell-cell adhesion prevents invasiveness of human carcinoma cells. *J. Cell Biol.*, **113**, 173–185.

FUKUYAMA R AND SIMIZU N. (1991). Detection of epidermal growth factor receptors and E-cadherin in the basolateral membrane of A431 cells by laser scanning fluorescence microscopy. *Jpn J. Cancer Res.*, **82**, 8–11.

GAVRILOVIC J, MOENS G, THIERY JP AND JOUANNEAU J. (1990). Expression of transfected transforming growth factor alpha induces a motile fibroblastlike phenotype with extracellular matrix-degrading potential in a rat bladder carcinoma cell line. *Cell Regulation*, **1**, 1003–1014.

HAMAGUCHI M, MATSUYOSHI N, OHNISHI Y, GOTOH B, TAKEICHI M AND NAGAI Y. (1993). p60v-src causes tyrosine phosphorylation and inactivation of the N-cadherin-catenin cell adhesion system. *EMBO J.*, **12**, 307–314.

IIHARA K, SHIOZAKI H, TAHARA H, INOUE M, KOBAYASHI K, TAMURA S, OKA H, MIYATA M, DOKI Y AND MORI T. (1993). Prognostic significance of transforming growth factor α in human esophageal carcinomas; implication for the autocrine proliferation. *Cancer*, **71**, 2902–2909.

ITOH M, YONEMURA S, NAGAFUCHI A, TSUKITA SA AND TSUKITA SH. (1991). A 220-kD undercoat-constitutive protein: its specific localization at cadherin-based cell–cell adhesion sites. *J. Cell Biol.*, **115**, 1449–1462.

KADOWAKI T, SHIOZAKI H, INOUE M, TAMURA S, OKA H, DOKI Y, IIHARA K, MATSUI S, IWAZAWA T, NAGAFUCHI A, TSUKITA S AND MORI T. (1994). E-cadherin and α-catenin expression in human esophageal cancer. *Cancer Res.*, **54**, 291–296.

LUND-JOHANSEN M, BJERKVIG R, HUMPHREY AP, BIGNER HS, BINGER DD AND LAERUM O. (1990). Effect of epidermal growth factor on glioma cell growth, migration, and invasion *in vitro*. *Cancer Res.*, **50**, 6039–6044.

MALDEN LT, NOVAK U, KAYE AH AND BURGESS AW. (1988). Selective amplification of the cytoplasmic domain of the epidermal growth factor receptor gene in glioblastoma multiforme. *Cancer Res.*, **48**, 2711–2714.

MATSUI S, SHIOZAKI H, INOUE M, TAMURA S, DOKI Y, KADOWAKI T, IWAZAWA T, SHIMAYA K, NAGAFUCHI A, TSUKITA S AND MORI T. (1994). Immunohistochemical evaluation of α-catenin expression in human gastric cancer. *Virchows Arch.*, **424**, 375–381.

MATSUMOTO K AND NAKAMURA T. (1992). Hepatocyte growth factor: molecular structure, roles in liver regeneration, and other biological functions. *Crit. Rev. Oncogen.*, **3**, 27–54.

MATSUYOSHI N, HAMAGUCHI M, TANIGUCHI S, NAGAFUCHI A, TSUKITA S AND TAKEICHI M. (1992). Cadherin-mediated cell–cell adhesion is perturbed by v-src tyrosine phosphorylation in metastatic fibroblasts. *J. Cell Biol.*, **118**, 703–714.

NAGAFUCHI A AND TAKEICHI M. (1988). Cell binding function of E-cadherin is regulated by the cytoplasmic domain. *EMBO J.*, **7**, 3679–3684.

NIEDBALA MJ AND SARTORELLI AC. (1989). Regulation by epidermal growth factor of human squamous cell carcinoma plasminogen activator-mediated proteolysis of extracellular matrix. *Cancer Res.*, **49**, 3302–3309.

NOSE A AND TAKEICHI M. (1986). A novel cadherin cell adhesion molecule: its expression patterns associated with implantation and organogenesis of mouse embryos. *J. Cell Biol.*, **103**, 2649–2658.

ODA T, KANAI Y, OYAMA T, YOSHIURA K, SHIMOYAMA Y, BIRCHMEIER W, SUGIMURA T AND HIROHASHI S. (1994). E-cadherin gene mutations in human gastric carcinoma cell lines. *Proc. Natl Acad. Sci. USA*, **91**, 1858–1862.

OKA H, SHIOZAKI H, KOBAYASHI K, INOUE M, TAHARA H, KOBAYASHI T, TAKATSUKA Y, MATUSYOSHI N, HIRANO S, TAKEICHI M AND MORI T. (1993). Expression of E-cadherin cell adhesion molecules in human breast cancer tissues and its relationship to metastasis. *Cancer Res.*, **53**, 1696–1701.

OZAWA M, BARIBAULT H AND KEMLER R. (1989). The cytoplasmic domain of the cell adhesion molecule uvomorulin associates with three independent proteins structurally related in different species. *EMBO J.*, **8**, 1711–1717.

RUBINFELD B, SOUZA B, ALBERT I, MÜLLER O, CHAMBERLAIN SH, MASIARZ FR, MUNEMITSU S AND POLAKIS P. (1993). Association of the APC gene product with β-catenin. *Science*, **262**, 1731–1734.

SAINSBURY JR, FARNDON JR, SHERBET GV AND HARRIS AL. (1985). Epidermal growth factor receptors and oestrogen receptors in human breast cancer. *Lancet*, **i**, 364–366.

SHIMOYAMA Y, HIROHASHI S, HIRANO S, NOGUCHI M, SHIMOSATO Y, TAKEICHI M AND ABE O. (1989). Cadherin cell-adhesion molecules in human epithelial tissues and carcinomas. *Cancer Res.*, **49**, 2128–2133.

SHIMOYAMA Y, NAGAFUCHI A, FUJITA S, GOTOH M, TAKEICHI M, TSUKITA S AND HIROHASHI S. (1992). Cadherin dysfunction in a human cancer cell line: possible involvement of loss of α-catenin expression in reduced cell–cell adhesiveness. *Cancer Res.*, **52**, 5770–5774.

SU L, VOGELSTEIN B AND KINZLER KW. (1993). Association of the APC tumor suppressor protein with catenins. *Science*, **262**, 1734–1737.

TAKEICHI M. (1977). Functional correlation between cell adhesive properties and some cell surface proteins. *J. Cell Biol.*, **75**, 464–474.

TAKESHIMA E, HAMAGUCHI M, WATANABE T, AKIYAMA S, KATAOKA M, OHNISHI Y, XIAO H, NAGAI Y AND TAKAGI H. (1991). Aberrant evaluation of tyrosine-specific phosphorylation in human gastric cancer cells. *Jpn J. Cancer Res.*, **82**, 1428–1435.

EUHARA Y, MURAKAMI Y, MIZUNO S AND KAWAI S. (1988). Inhibition of transforming activity of tyrosine kinase oncogenes by herbimycin A. *Virology*, **164**, 294–298.

ULRICH A AND SCHLESSINGER J. (1990). Signal transduction by receptors with tyrosine kinase activity. *Cell*, **61**, 203–212.

YOSHIDA K, TSUDA T, MATSUMURA T, TSUJINO T, HATTORI T, ITO H AND TAHARA E. (1989). Amplification of epidermal growth factor receptor (EGFR) gene and oncogenes in human gastric carcinomas. *Virchows Arch [B]*, **57**, 285–290.


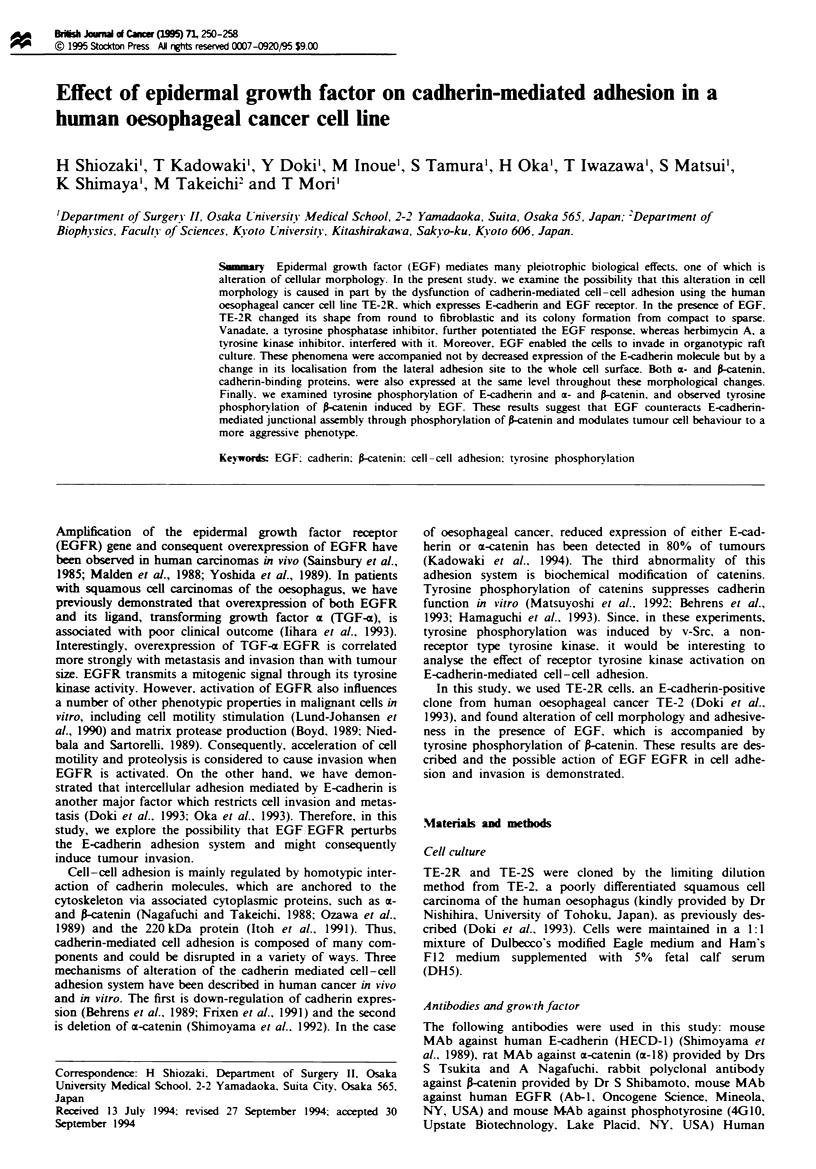

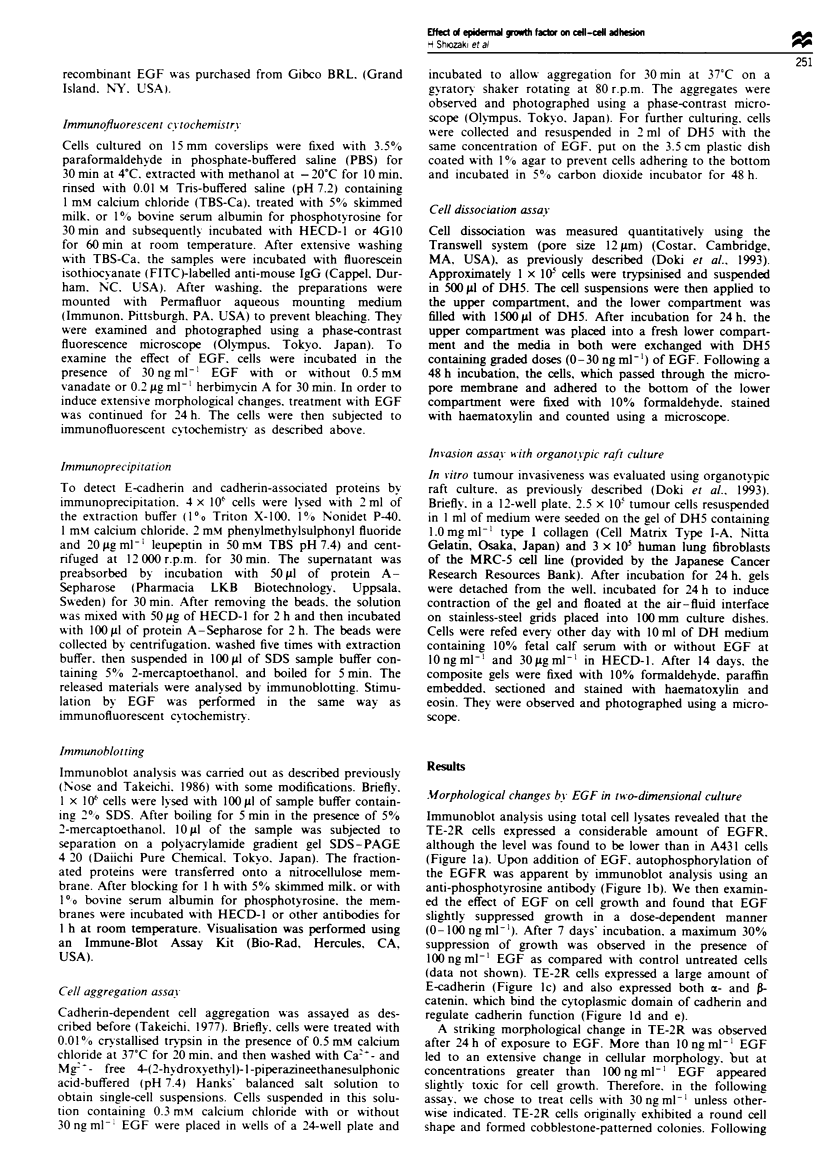

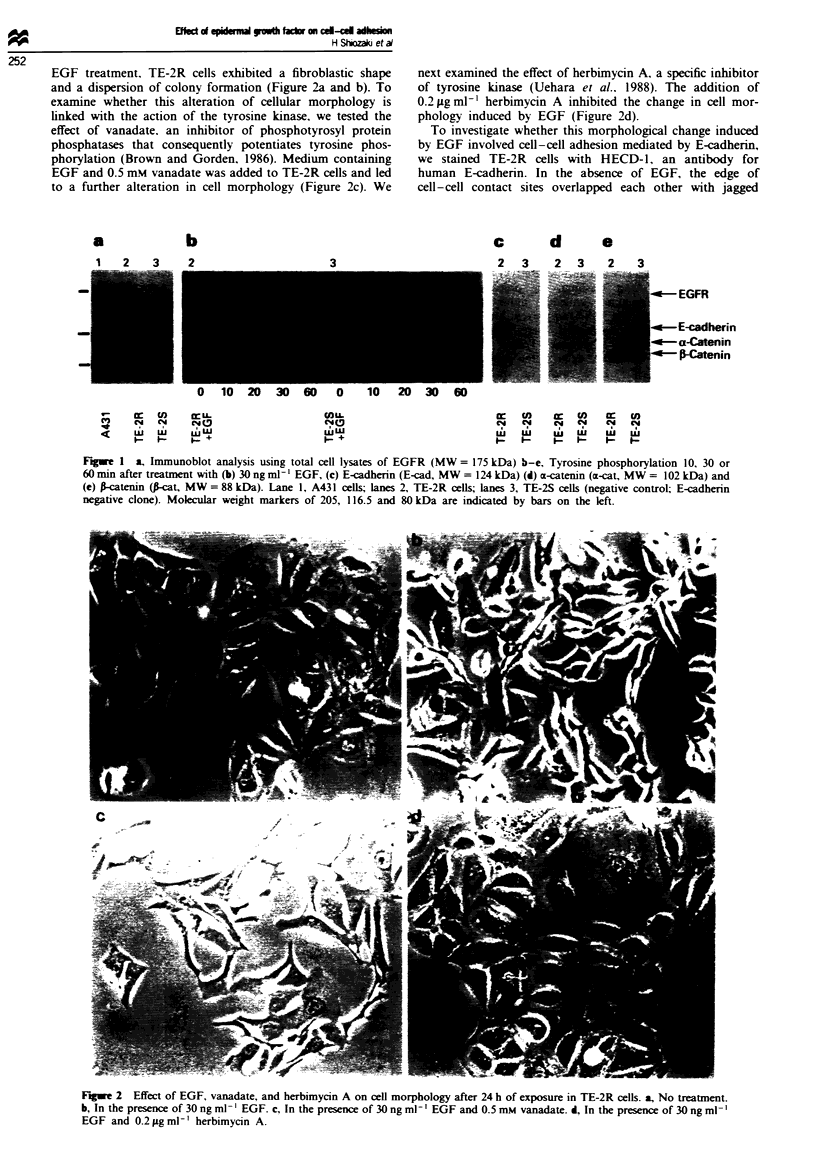

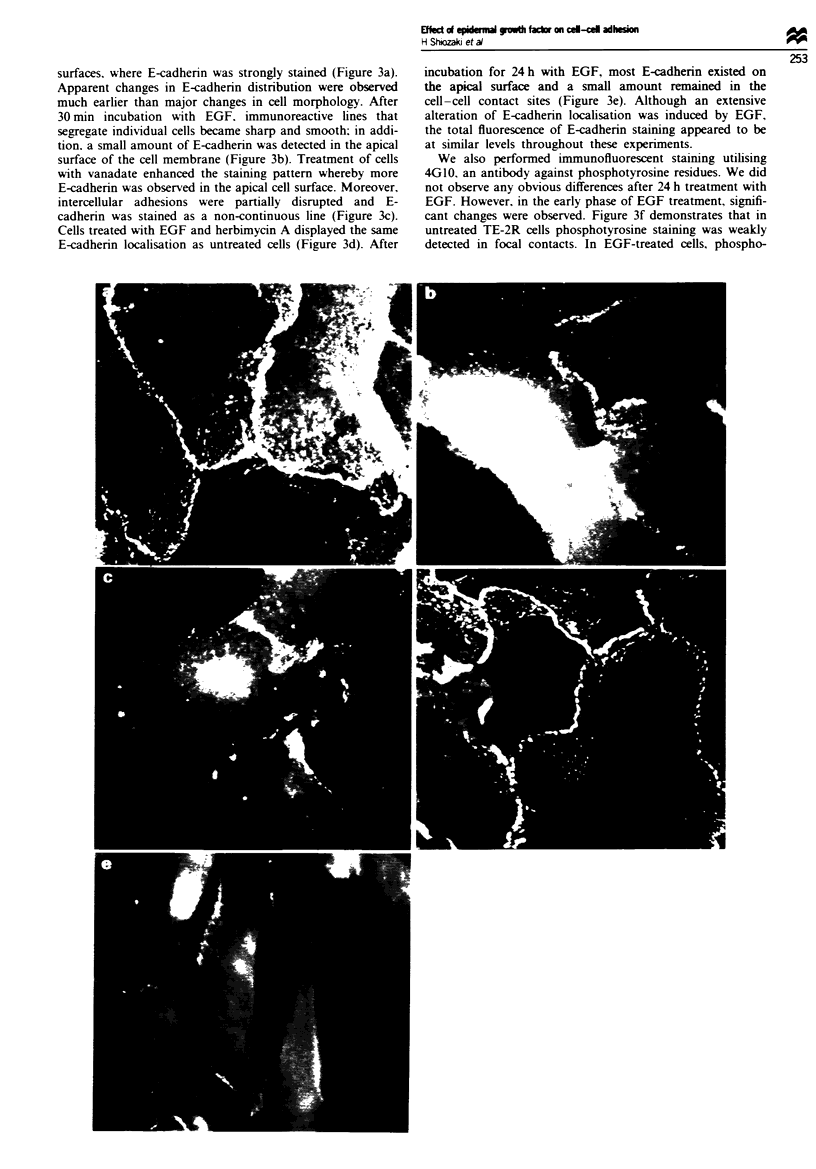

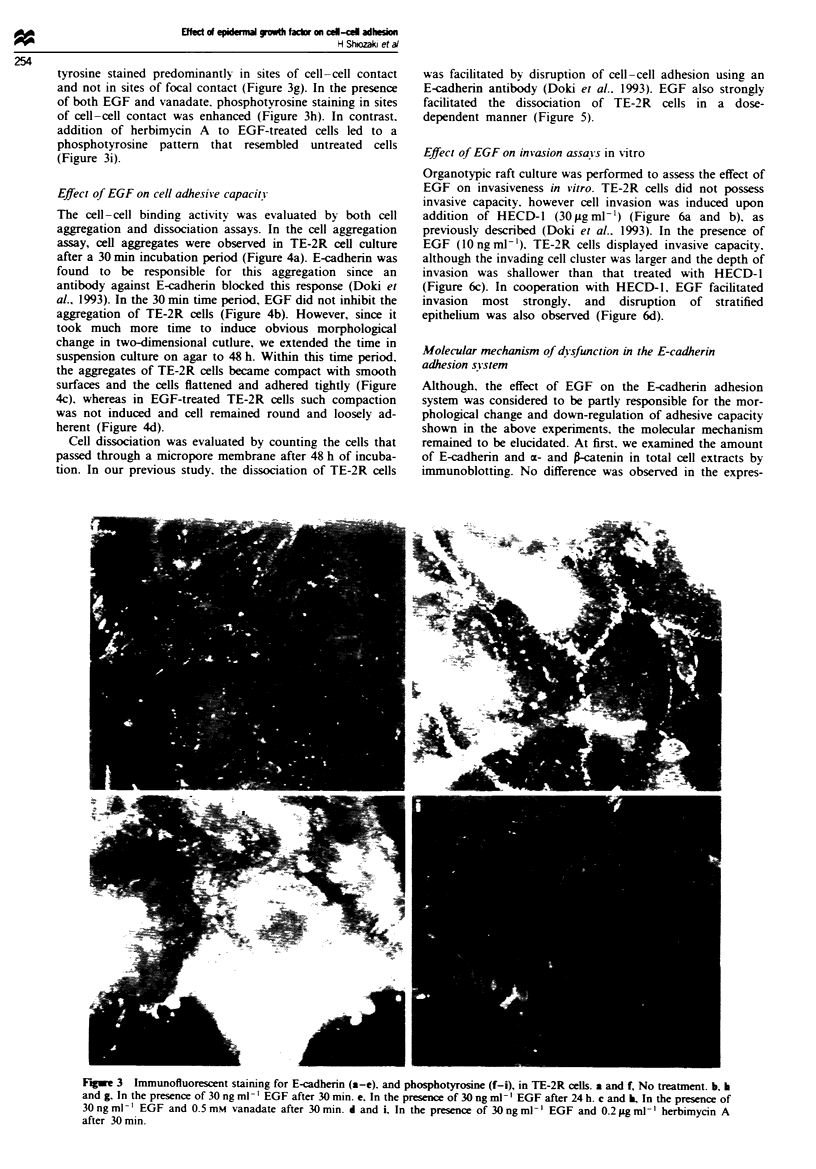

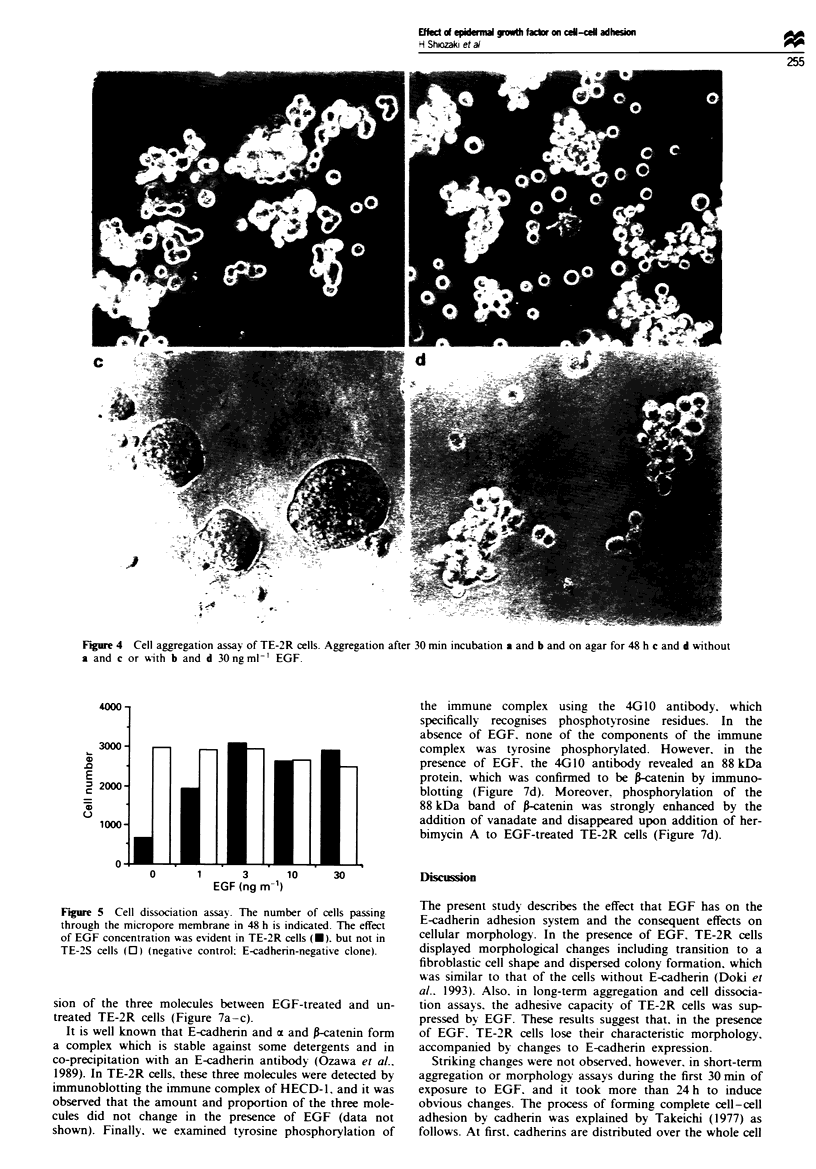

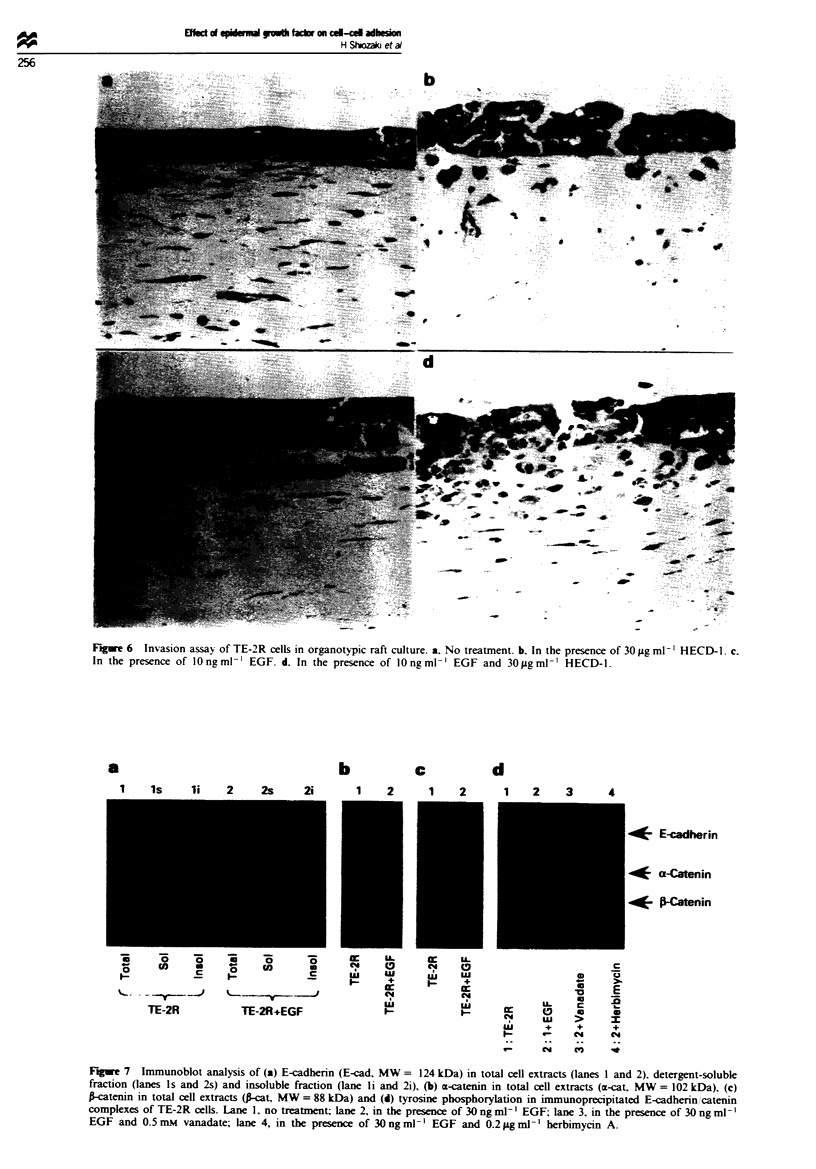

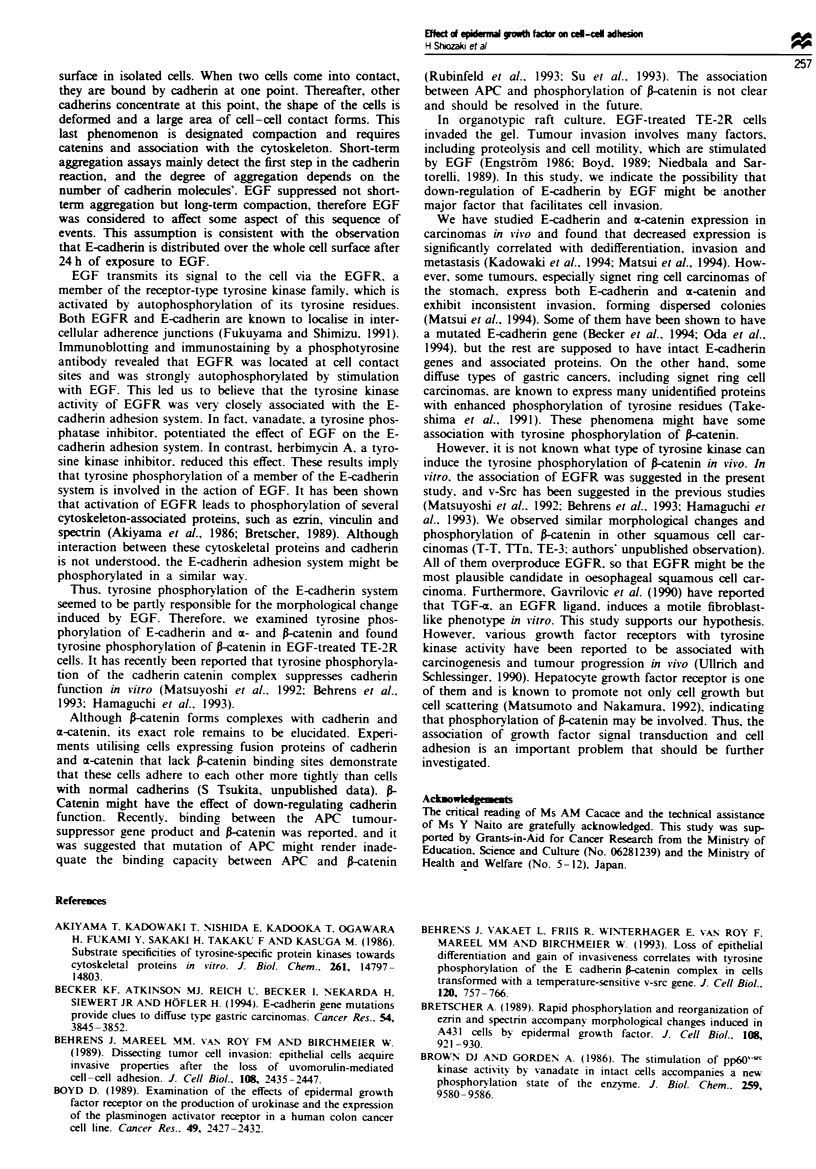

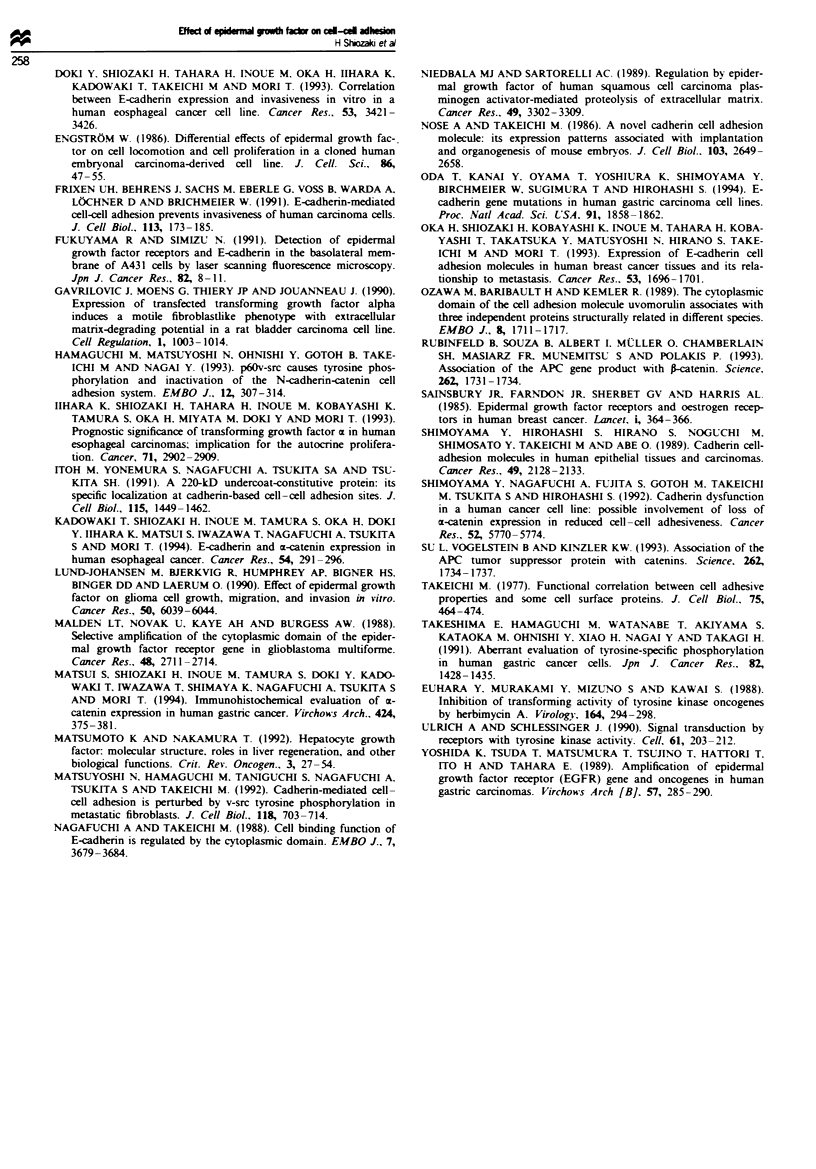


## References

[OCR_00702] Akiyama T., Kadowaki T., Nishida E., Kadooka T., Ogawara H., Fukami Y., Sakai H., Takaku F., Kasuga M. (1986). Substrate specificities of tyrosine-specific protein kinases toward cytoskeletal proteins in vitro.. J Biol Chem.

[OCR_00708] Becker K. F., Atkinson M. J., Reich U., Becker I., Nekarda H., Siewert J. R., Höfler H. (1994). E-cadherin gene mutations provide clues to diffuse type gastric carcinomas.. Cancer Res.

[OCR_00714] Behrens J., Mareel M. M., Van Roy F. M., Birchmeier W. (1989). Dissecting tumor cell invasion: epithelial cells acquire invasive properties after the loss of uvomorulin-mediated cell-cell adhesion.. J Cell Biol.

[OCR_00724] Behrens J., Vakaet L., Friis R., Winterhager E., Van Roy F., Mareel M. M., Birchmeier W. (1993). Loss of epithelial differentiation and gain of invasiveness correlates with tyrosine phosphorylation of the E-cadherin/beta-catenin complex in cells transformed with a temperature-sensitive v-SRC gene.. J Cell Biol.

[OCR_00718] Boyd D. (1989). Examination of the effects of epidermal growth factor on the production of urokinase and the expression of the plasminogen activator receptor in a human colon cancer cell line.. Cancer Res.

[OCR_00732] Bretscher A. (1989). Rapid phosphorylation and reorganization of ezrin and spectrin accompany morphological changes induced in A-431 cells by epidermal growth factor.. J Cell Biol.

[OCR_00741] Brown D. J., Gordon J. A. (1984). The stimulation of pp60v-src kinase activity by vanadate in intact cells accompanies a new phosphorylation state of the enzyme.. J Biol Chem.

[OCR_00751] Doki Y., Shiozaki H., Tahara H., Inoue M., Oka H., Iihara K., Kadowaki T., Takeichi M., Mori T. (1993). Correlation between E-cadherin expression and invasiveness in vitro in a human esophageal cancer cell line.. Cancer Res.

[OCR_00757] Engström W. (1986). Differential effects of epidermal growth factor (EGF) on cell locomotion and cell proliferation in a cloned human embryonal carcinoma-derived cell line in vitro.. J Cell Sci.

[OCR_00764] Frixen U. H., Behrens J., Sachs M., Eberle G., Voss B., Warda A., Löchner D., Birchmeier W. (1991). E-cadherin-mediated cell-cell adhesion prevents invasiveness of human carcinoma cells.. J Cell Biol.

[OCR_00769] Fukuyama R., Shimizu N. (1991). Detection of epidermal growth factor receptors and E-cadherins in the basolateral membrane of A431 cells by laser scanning fluorescence microscopy.. Jpn J Cancer Res.

[OCR_00775] Gavrilović J., Moens G., Thiery J. P., Jouanneau J. (1990). Expression of transfected transforming growth factor alpha induces a motile fibroblast-like phenotype with extracellular matrix-degrading potential in a rat bladder carcinoma cell line.. Cell Regul.

[OCR_00783] Hamaguchi M., Matsuyoshi N., Ohnishi Y., Gotoh B., Takeichi M., Nagai Y. (1993). p60v-src causes tyrosine phosphorylation and inactivation of the N-cadherin-catenin cell adhesion system.. EMBO J.

[OCR_00789] Iihara K., Shiozaki H., Tahara H., Kobayashi K., Inoue M., Tamura S., Miyata M., Oka H., Doki Y., Mori T. (1993). Prognostic significance of transforming growth factor-alpha in human esophageal carcinoma. Implication for the autocrine proliferation.. Cancer.

[OCR_00795] Itoh M., Yonemura S., Nagafuchi A., Tsukita S., Tsukita S. (1991). A 220-kD undercoat-constitutive protein: its specific localization at cadherin-based cell-cell adhesion sites.. J Cell Biol.

[OCR_00801] Kadowaki T., Shiozaki H., Inoue M., Tamura S., Oka H., Doki Y., Iihara K., Matsui S., Iwazawa T., Nagafuchi A. (1994). E-cadherin and alpha-catenin expression in human esophageal cancer.. Cancer Res.

[OCR_00808] Lund-Johansen M., Bjerkvig R., Humphrey P. A., Bigner S. H., Bigner D. D., Laerum O. D. (1990). Effect of epidermal growth factor on glioma cell growth, migration, and invasion in vitro.. Cancer Res.

[OCR_00813] Malden L. T., Novak U., Kaye A. H., Burgess A. W. (1988). Selective amplification of the cytoplasmic domain of the epidermal growth factor receptor gene in glioblastoma multiforme.. Cancer Res.

[OCR_00819] Matsui S., Shiozaki H., Inoue M., Tamura S., Doki Y., Kadowaki T., Iwazawa T., Shimaya K., Nagafuchi A., Tsukita S. (1994). Immunohistochemical evaluation of alpha-catenin expression in human gastric cancer.. Virchows Arch.

[OCR_00824] Matsumoto K., Nakamura T. (1992). Hepatocyte growth factor: molecular structure, roles in liver regeneration, and other biological functions.. Crit Rev Oncog.

[OCR_00832] Matsuyoshi N., Hamaguchi M., Taniguchi S., Nagafuchi A., Tsukita S., Takeichi M. (1992). Cadherin-mediated cell-cell adhesion is perturbed by v-src tyrosine phosphorylation in metastatic fibroblasts.. J Cell Biol.

[OCR_00837] Nagafuchi A., Takeichi M. (1988). Cell binding function of E-cadherin is regulated by the cytoplasmic domain.. EMBO J.

[OCR_00840] Niedbala M. J., Sartorelli A. C. (1989). Regulation by epidermal growth factor of human squamous cell carcinoma plasminogen activator-mediated proteolysis of extracellular matrix.. Cancer Res.

[OCR_00846] Nose A., Takeichi M. (1986). A novel cadherin cell adhesion molecule: its expression patterns associated with implantation and organogenesis of mouse embryos.. J Cell Biol.

[OCR_00854] Oda T., Kanai Y., Oyama T., Yoshiura K., Shimoyama Y., Birchmeier W., Sugimura T., Hirohashi S. (1994). E-cadherin gene mutations in human gastric carcinoma cell lines.. Proc Natl Acad Sci U S A.

[OCR_00861] Oka H., Shiozaki H., Kobayashi K., Inoue M., Tahara H., Kobayashi T., Takatsuka Y., Matsuyoshi N., Hirano S., Takeichi M. (1993). Expression of E-cadherin cell adhesion molecules in human breast cancer tissues and its relationship to metastasis.. Cancer Res.

[OCR_00867] Ozawa M., Baribault H., Kemler R. (1989). The cytoplasmic domain of the cell adhesion molecule uvomorulin associates with three independent proteins structurally related in different species.. EMBO J.

[OCR_00874] Rubinfeld B., Souza B., Albert I., Müller O., Chamberlain S. H., Masiarz F. R., Munemitsu S., Polakis P. (1993). Association of the APC gene product with beta-catenin.. Science.

[OCR_00879] Sainsbury J. R., Farndon J. R., Sherbet G. V., Harris A. L. (1985). Epidermal-growth-factor receptors and oestrogen receptors in human breast cancer.. Lancet.

[OCR_00884] Shimoyama Y., Hirohashi S., Hirano S., Noguchi M., Shimosato Y., Takeichi M., Abe O. (1989). Cadherin cell-adhesion molecules in human epithelial tissues and carcinomas.. Cancer Res.

[OCR_00891] Shimoyama Y., Nagafuchi A., Fujita S., Gotoh M., Takeichi M., Tsukita S., Hirohashi S. (1992). Cadherin dysfunction in a human cancer cell line: possible involvement of loss of alpha-catenin expression in reduced cell-cell adhesiveness.. Cancer Res.

[OCR_00897] Su L. K., Vogelstein B., Kinzler K. W. (1993). Association of the APC tumor suppressor protein with catenins.. Science.

[OCR_00902] Takeichi M. (1977). Functional correlation between cell adhesive properties and some cell surface proteins.. J Cell Biol.

[OCR_00905] Takeshima E., Hamaguchi M., Watanabe T., Akiyama S., Kataoka M., Ohnishi Y., Xiao H. Y., Nagai Y., Takagi H. (1991). Aberrant elevation of tyrosine-specific phosphorylation in human gastric cancer cells.. Jpn J Cancer Res.

[OCR_00912] Uehara Y., Murakami Y., Mizuno S., Kawai S. (1988). Inhibition of transforming activity of tyrosine kinase oncogenes by herbimycin A.. Virology.

[OCR_00917] Ullrich A., Schlessinger J. (1990). Signal transduction by receptors with tyrosine kinase activity.. Cell.

[OCR_00921] Yoshida K., Tsuda T., Matsumura T., Tsujino T., Hattori T., Ito H., Tahara E. (1989). Amplification of epidermal growth factor receptor (EGFR) gene and oncogenes in human gastric carcinomas.. Virchows Arch B Cell Pathol Incl Mol Pathol.

